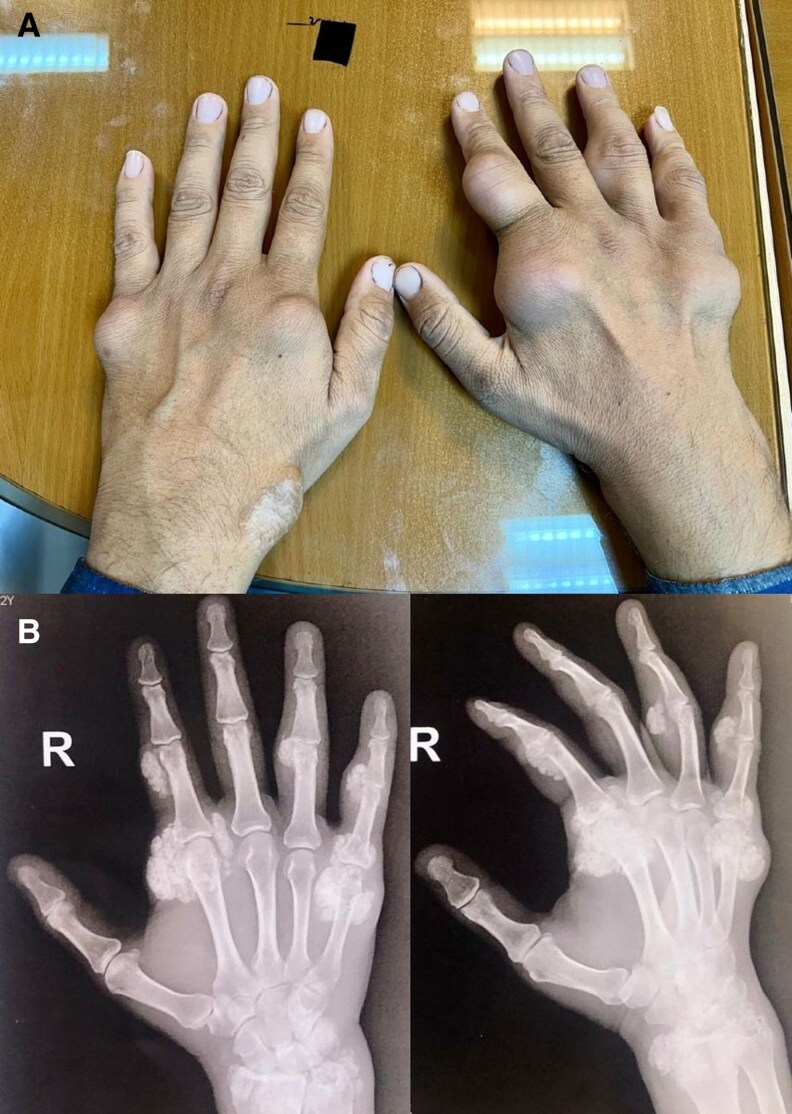# Tumoral calcinosis in a patient with end-stage renal disease

**DOI:** 10.1210/jcemcr/luag142

**Published:** 2026-06-01

**Authors:** Amir Bahrami, Hamidreza Ashayeri, Mahsa Malekian

**Affiliations:** Endocrine Research Centre, Tabriz University of Medical Sciences, Tabriz 5166614766, Iran; Research Center for Evidence-Based Medicine, Iranian EBM Centre: A JBI Centre of Excellence, Faculty of Medicine, Tabriz University of Medical Sciences, Tabriz 516615731, Iran; Endocrine Research Centre, Tabriz University of Medical Sciences, Tabriz 5166614766, Iran

**Keywords:** tumoral calcinosis, renal secondary hyperparathyroidism, hyperphosphatemia

## Image legend

A 32-year-old man presented with painless, subcutaneous nodules that had developed during the previous 4 months, in his hands (Panel A). His medical history was notable for hypertension leading to end-stage renal disease and hemodialysis for 18 months. A plain film of the patient's hands showed amorphous multilobulated calcified masses in the wrist, metacarpophalangeal, and proximal interphalangeal periauricular regions (Panel B). Laboratory tests revealed severe secondary hyperparathyroidism with an intact parathyroid hormone (iPTH) of 1732 pg/mL (SI: 1732 ng/L) (normal range: 15-68.3 pg/mL [SI: 15-68.3 ng/L]), phosphorus of 7.7 mg/dL (SI: 2.49 mmol/L) (normal range: 2.6-4.5 mg/dL [SI: 0.84-1.45 mmol/L]), and calcium of 8.9 mg/dL (SI: 2.23 mmol/L) (normal range: 8.5-10.5 mg/dL [SI: 2.13-2.63 mmol/L]). Ultrasound showed two asymmetrical enlarged parathyroid glands, suggesting parathyroid hyperplasia as the cause of secondary hyperparathyroidism. Medical attempts to lower PTH were unsuccessful, and the patient was considered for parathyroidectomy.

**Figure:**